# Roxadustat and incidence of peritoneal dialysis-associated peritonitis: a propensity score–matched cohort study

**DOI:** 10.1007/s10157-026-02926-6

**Published:** 2026-08-10

**Authors:** Ya-Hui Yang, Yang Zhao, Wen Tang

**Affiliations:** 1 Department of Nephrology, Peking University Third Hospital, 49 North Garden Rd, Haidian District, Beijing, 100191 P. R. China; 2 Department of Laboratory Medicine, Peking University Third Hospital, Beijing, P. R. China

**Keywords:** Roxadustat, Peritoneal dialysis, Peritonitis, Propensity score matching, HIF-PHI, Erythropoietin

## Abstract

**Background:**

Roxadustat, a hypoxia-inducible factor prolyl hydroxylase inhibitor (HIF-PHI), exerts pleiotropic effects beyond erythropoiesis, including modulation of inflammation and immune responses. Preliminary studies suggest Roxadustat may modulate inflammation to reduce peritoneal dialysis-associated peritonitis (PDAP) risk, but this requires further proof.

**Methods:**

Patients treated with Roxadustat or recombinant human erythropoietin (rhuEPO) at least three months from September 2014 and September 2024 were respectively enrolled. 1:1 propensity score matching (PSM) was performed based on age, sex, albumin, hemoglobin, lipid profiles, and initial urine volume. The primary outcome was PDAP incidence rate, expressed as episodes per person-year. Incidence rate ratios (IRRs) were estimated using Poisson regression models.

**Results:**

Among 296 eligible patients, PSM yielded 136 well-balanced individuals (68 per group). Over 225.85 patient-years of follow-up, 41 peritonitis episodes were recorded. The Roxadustat group exhibited a significantly lower PDAP incidence rate than the rhuEPO group (0.131 vs. 0.212 episodes per patient-year). Multivariable Poisson regression, adjusted for age, potassium, and albumin, confirmed that Roxadustat was associated with lower PDAP risk (adjusted IRR 0.494, 95% CI 0.252–0.970; *p* = 0.041). The trend persisted after multivariate adjustment.

**Conclusions:**

Roxadustat was associated with a reduced incidence of PDAP compared to rhuEPO, suggesting that HIF stabilization may offer protective benefits beyond anemia correction. Prospective validation is warranted.

**Supplementary Information:**

The online version contains supplementary material available at https://doi.org/10.1007/s10157-026-02926-6.

## Introduction

Peritoneal dialysis (PD) is a widely used renal replacement therapy for patients with end-stage kidney disease (ESKD) [1]. Peritoneal dialysis-associated peritonitis (PDAP) remains the leading cause of technique failure, transfer to hemodialysis, and mortality [2]. Factors associated with PDAP are multifactorial while recent study implied that Roxadustat may modulate inflammation to reduce PDAP risk [3–6] .

Roxadustat has been introduced as a novel treatment for renal anemia [7]. Beyond erythropoiesis, hypoxia-inducible factor (HIF) stabilization improves iron homeostasis, lowers hepcidin, and modulates inflammatory pathways [8]. HIF-1 activation has also been shown to enhance innate immune responses, support epithelial barrier integrity [9], and regulate antimicrobial defense [10, 11] —all mechanisms may relevant to the susceptibility and progression of PDAP. Experimental data demonstrate that Roxadustat can attenuate peritoneal inflammation and fibrosis [12], while early clinical observations suggest favorable effects on gut microbiota composition and systemic inflammation [13] .

Despite this biological plausibility, clinical evidence remains sparse. Although afore mentioned preliminary studies suggest Roxadustat may modulate inflammation to reduce PDAP risk [6], yet definitive, comparative real-world data is still lacking due to existing studies’ heterogeneous treatment histories (notably patients switching from rhuEPO), a factor that obscures the drug’s independent effects. To address this, we evaluated the specific impact of Roxadustat monotherapy on the risk of PDAP.

## Methods

### Study participants and design

We included patients initiating PD at Peking University Third Hospital between September 2014 and September 2024. The study was divided into Phase 1 (Sep 2014–Aug 2019; rhuEPO only) and Phase 2 (Sep 2019–Aug 2024; Roxadustat introduced). Follow-up concluded on Aug 31, 2019, for Phase 1 and Aug 31, 2024, for Phase 2.

Eligible patients were ≥ 18 years old, diagnosed with CKD stage 5, on regular PD for ≥ 90 days, and received ≥ 3 months of Roxadustat or rhuEPO monotherapy. Exclusion criteria included combination therapy, NYHA Class III-IV heart failure, recent cardiovascular events, cirrhosis, malignancy, or active autoimmune diseases.

Patients were assigned to the Roxadustat (*n* = 90) or rhuEPO (*n* = 206) group based on their initial medication recorded in the electronic medical system (the “first medication” principle). Those receiving combination therapy or switching between Roxadustat and rhuEPO during the study were excluded.

Roxadustat was dosed thrice weekly (100 mg for < 60 kg; 120 mg for ≥ 60 kg). rhuEPO was administered subcutaneously at 80–120 U/kg/week. Both regimens were titrated to maintain hemoglobin between 10 and 12 g/dL. All patients received standardized PD training from a multidisciplinary clinical team.

### Data collection, measurements, and follow-up

Patients were prospectively followed from the date of PD catheter insertion. Demographic, clinical, and laboratory data were retrospectively extracted from electronic medical records by a specialized clinical team; initial urine volume and all baseline laboratory parameters were obtained at the time of PD initiation. Standardized follow-up occurred every 1–3 months, including physical examinations, biochemical assessments, and PD regimen reviews. Serum albumin and other biochemical parameters were measured using standard laboratory methods. Residual renal function (RRF) was assessed at baseline and monitored during follow-up, quantified as the residual glomerular filtration rate (rGFR) calculated by averaging the 24-hour urea and creatinine clearances [14]; the rGFR and its corresponding residual urine volume were assessed around three months after PD initiation. Dialysis dose was defined as the prescribed instilled dialysate volume. Dialysis adequacy was primarily assessed by total weekly urea(Kt/V) [15] and total Creatinine Clearance (tCCr) [16], with total Kt/V incorporating both peritoneal and residual renal contributions; the urea distribution volume was estimated using Watson’s formula [17]. PD modality, prescribed PD dose, and connection system (automated cycler system or manual twin-bag system) were recorded from PD regimen documentation. Follow-up continued until death, renal transplantation, transfer to hemodialysis or another center, PD discontinuation, or study termination (Aug 31, 2019/2024); each peritonitis episode was recorded as an independent recurrent outcome event and contributed to the event count in the Poisson regression analysis, without terminating follow-up.

### Study outcome and peritonitis definition

PDAP was diagnosed according to the International Society for Peritoneal Dialysis (ISPD) guidelines [18]. The diagnosis required at least two of the following criteria: (1) Clinical features of abdominal pain and/or cloudy peritoneal effluent; (2) Dialysate white cell count > 100 cells/µL after a dwell time of at least 2 h with > 50% polymorphonuclear leukocytes; and (3) Positive dialysate effluent culture. The primary outcome, PDAP incidence, was calculated as the number of peritonitis episodes per patient-year of follow-up. Microbiological profiles were recorded to compare pathogen distribution between groups.

### Statistical analysis

Baseline characteristics were compared using two-sample t-tests for normally distributed continuous data (mean ± SD), Mann-Whitney U tests (or medians [IQR]) for non-normal data, and chi-square tests for categorical variables.

To minimize baseline confounding, 1:1 propensity score matching (PSM) was performed using nearest-neighbor matching with a caliper width of 0.2. Matching variables, using data at the time of PD initiation, included age, sex, hemoglobin, albumin, initial urine volume, and lipid profiles (TC, TG, LDL-C, and HDL-C), which initially showed significant imbalances (*p* < 0.05, except for age and sex). This procedure yielded two well-balanced, comparable cohorts. Balance after matching was assessed using the standardized mean difference (SMD), with an SMD < 0.1 indicating balance and an SMD < 0.2 considered acceptable, consistent with established recommendations for propensity-score-matched analyses; all covariates achieved an SMD < 0.1 in the matched cohort.

Crude incidence rates (episodes per person-year) and 95% CIs were calculated using the Garwood method (Poisson distribution). Post-matching, rates and 95% CIs were estimated via pairwise Poisson regression with robust sandwich standard errors, treating matched pairs as clusters and log(person-year) as an offset. Univariate Poisson regression (with log-offset for observation time) was used for initial screening. Variables with *p* < 0.05 or clinical relevance were entered into a multivariable Poisson model to calculate adjusted incidence rate ratios (IRRs) and 95% CIs.

Statistical analyses were performed using SPSS v26.0 (IBM, Armonk, NY, USA) and R v4.4.2 (http://www.R-project.org/). A two-sided p-value < 0.05 was considered statistically significant.

## Results

### Characteristics of study participants

**Fig. 1:**
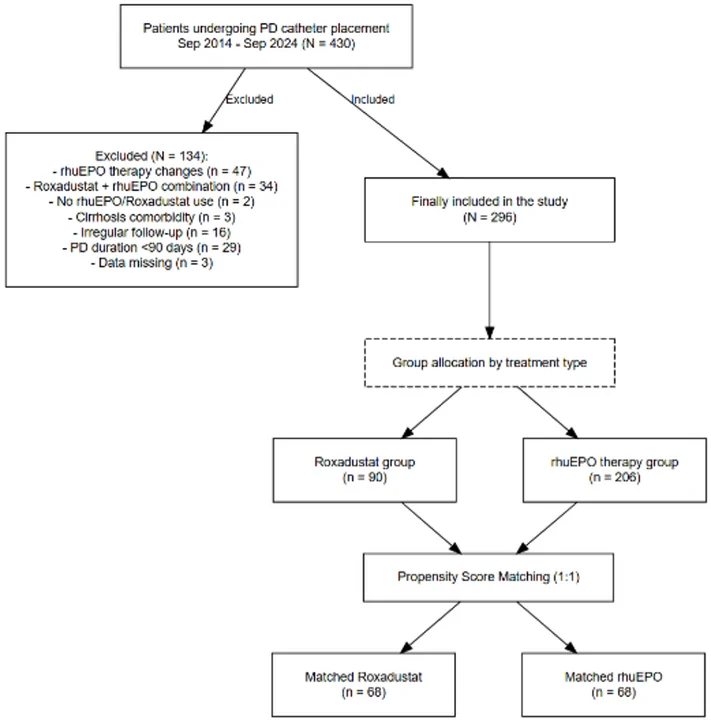
Flow chart of study participants. *PD* peritoneal dialysis, *rhuEPO* recombinant human erythropoietin

A total of 296 PD patients were included in the unmatched cohort (Roxadustat, *n* = 90; rhuEPO, *n* = 206; Fig. 1). The mean age of the cohort was 58.28 ± 16.71 years, and 194 (65.5%) were male. Prior to PSM, the Roxadustat group exhibited lower levels of albumin (*p* < 0.001), triglycerides (*p* = 0.037), total cholesterol (*p* < 0.001), low-density lipoprotein (LDL) (*p* < 0.001), high-density lipoprotein (HDL) (*p* < 0.001), initial urine volume (*p* = 0.016), higher automated peritoneal dialysis (APD) usage (23.3% vs. 9.2%, *p* = 0.002), and elevated Transferrin Saturation (TSAT) (*p* = 0.046), while diabetes prevalence, Kt/V, high-sensitivity C-Reactive Protein (hsCRP), and serum ferritin(SF) were comparable between groups (Table 1).

**Table 1:** Baseline characteristics of study participants at the initiation of peritoneal dialysis, by treatment group, before and after propensity score matching

Variables	All			After matching			
	Roxadustat group *N* = 90	rhuEPO group *N* = 206	*p* value	Roxadustat group *N* = 68		rhuEPO group *N* = 68	*p* value
Age, years	60.23 ± 16.73	57.43 ± 16.67	0.188	59.03 ± 16.20		59.55 ± 15.38	0.850
Male, n (%)	66(73.3%)	128(62.1%)	0.062	49(72.1%)		47(69.1%)	0.851
Primary disease, n (%)			0.026				0.207
DN, n (%)	40 (44.4%)	88 (42.7%)	—	31 (45.6%)		38 (55.9%)	—
GN, n (%)	20 (22.2%)	74 (35.9%)	—	15 (22.1%)		17 (25.0%)	—
Others, n (%)	30 (33.3%)	44 (21.4%)	—	22 (32.4%)		13 (19.1%)	—
Diabetes, n (%)	45(50.0%)	99(48.1%)	0.785	33(48.5%)		42(61.8%)	0.168
SBP, mmHg	149.02 ± 20.20	147.91 ± 37.05	0.281	147.31 ± 19.05		143.68 ± 55.84	0.555
DBP, mmHg	80.70 ± 13.55	81.93 ± 14.78	0.582	81.88 ± 13.93		81.51 ± 13.87	0.878
Initial urine volume, mL /24 h	1122.89 ± 673.71	1281.07 ± 634.54	0.016	1134.41 ± 679.72		1182.79 ± 672.97	0.563
dialysis dose	7868.82 ± 1589.16	7948.56 ± 1570.93	0.613	7873.81 ± 1581.45		8192.26 ± 1694.00	0.331
Total Kt/V	1.98 ± 0.49	1.89 ± 0.42	0.133	1.98 ± 0.49		1.86 ± 0.43	0.076
Total Creatinine Clearance	81.75 ± 32.50	73.58 ± 26.03	0.024	82.78 ± 34.01		74.22 ± 27.02	0.061
Ultrafiltration Volume, ml	577.99 ± 408.51	599.89 ± 487.50	0.751	535.24 ± 395.75		599.89 ± 487.50	0.472
Residual GFR, mL/min	1.53 (0.00, 3.63)	0.74 (0.00, 4.82)	0.611	1.53 (0.04, 3.37)		0.80 (0.00, 6.17)	0.676
Residual urine volume, mL/24h	444.13 ± 404.92	389.13 ± 472.51	0.059	470.18 ± 417.85		362.7 ± 448.81	0.036
APD, n (%)	21 (23.3%)	19 (9.2%)	0.002	16 (23.5%)		9 (13.2%)	0.184
Serum creatinine, umol/L	747.24 ± 253.17	781.27 ± 295.42	0.387	763.24 ± 249.95		761.09 ± 334.62	0.591
Serum urea, mmol/L	29.40 ± 9.94	28.38 ± 9.56	0.372	29.65 ± 9.37		28.02 ± 10.13	0.114
Serum Albumin, g/L	34.60 ± 4.80	37.55 ± 5.20	<0.001	35.47 ± 4.58		35.33 ± 4.54	0.858
Hemoglobin, g/L	92.91 ± 16.78	87.19 ± 17.65	0.011	91.24 ± 16.16		91.05 ± 17.19	0.936
Serum calcium, mmol/L	2.03 ± 0.27	2.04 ± 0.29	0.928	2.04 ± 0.27		2.06 ± 0.30	0.682
Serum phosphorus, mmol/L	1.97 ± 0.55	1.94 ± 0.60	0.562	1.98 ± 0.54		1.91 ± 0.60	0.444
iPTH, pg/mL	274.15 ± 208.66	310.71 ± 240.68	0.208	270.52 ± 206.92		260.91 ± 196.74	0.845
Total bile acids, umol/L	2.96 ± 2.23	3.16 ± 3.29	0.589	2.61 ± 1.35		3.18 ± 3.48	0.573
Triglycerides, mmol/L	1.43 ± 0.74	1.61 ± 0.76	0.037	1.50 ± 0.75		1.47 ± 0.73	0.948
Total cholesterol, mmol/L	3.53 ± 0.98	4.32 ± 1.24	<0.001	3.67 ± 1.00		3.75 ± 0.97	0.689
LDL, mmol/L	1.96 ± 0.76	2.58 ± 0.94	<0.001	2.04 ± 0.79		2.11 ± 0.72	0.511
HDL, mmol/L	0.85 ± 0.25	1.03 ± 0.34	<0.001	0.89 ± 0.26		0.89 ± 0.24	0.875
Fasting glucose, mmol/L	5.53 ± 2.15	5.73 ± 2.90	0.393	5.52 ± 2.07		5.79 ± 2.12	0.411
ALT, IU/L	13.55 ± 11.67	13.23 ± 10.63	0.906	14.70 ± 12.87		12.06 ± 8.77	0.319
AST, IU/L	17.51 ± 8.54	16.62 ± 7.44	0.349	17.06 ± 7.20		16.34 ± 7.06	0.418
Total Bilirubin, umol/L	7.24 ± 2.82	6.96 ± 2.70	0.525	7.24 ± 2.74		6.59 ± 2.35	0.263
Direct Bilirubin, umol/L	1.64 ± 1.15	1.66 ± 1.06	0.954	1.52 ± 0.81		1.61 ± 1.06	0.941
Potassium, mmol/L	4.41 ± 0.69	4.53 ± 0.77	0.159	4.42 ± 0.73		4.57 ± 0.72	0.115
Sodium, mmol/L	138.90 ± 3.75	137.98 ± 10.40	0.795	138.84 ± 3.86		136.11 ± 17.11	0.969
Serum chloride, mmol/L	104.11 ± 5.90	104.48 ± 5.26	0.607	104.17 ± 5.74		105.60 ± 4.93	0.115
hsCRP, mg/L	2.31 (0.76, 9.98)	2.00(0.65, 6.49)	0.224	2.30(0.67, 6.04)		2.08(0.7, 8.12)	0.998
TSAT, %	22.37(15.54, 30.63)	19.54(13.67, 28.52)	0.046	21.32(15.93, 30.12)		19.57(15.34, 27.64)	0.258
SF, ug/L	141.05(77.62, 234.93)	156.00(62.90, 312.85)	0.617	129.00(74.25, 225)		185.80(98.83, 366.5)	0.021
Peritonitis episodes, n	18	55	—	12		29	—
Total patient-years of follow-up	139.87	406.12	—	101.38		124.47	—
Peritonitis rates, episodes per person-year	0.129	0.135	0.850	0.131		0.212	0.049
Causative Organisms, n (%)							0.567
Gram-positive, n (%)	8 (44.4%)	26 (47.3%)	—	5 (41.7%)		12 (41.4%)	—
Gram-negative, n (%)	6 (33.3%)	11 (20.0%)	—	4 (33.3%)		7 (24.1%)	—
Fungal, n (%)	1 (5.6%)	2 (3.6%)	—	—		1 (3.5%)	—
Polymicrobial, n (%)	—	1 (1.8%)	—	—		1 (3.5%)	—
Culture-negative, n (%)	3 (16.7%)	15 (27.3%)	—	3 (25.0%)		8 (27.5%)	—

Data are mean (SD), n (%), or median (IQR), unless stated otherwise

Primary disease categories were defined as follows: (1) DN included diabetic nephropathy and diabetic kidney disease; (2) GN included chronic glomerulonephritis, IgA nephropathy, and focal proliferative glomerulonephritis; (3) Others included hypertensive nephrosclerosis, ischemic nephropathy, polycystic kidney disease, lupus nephritis, interstitial nephritis, vasculitis, Alport syndrome, and cases with unknown etiology

*rhuEPO* recombinant human erythropoietin, *DN* diabetes nephropathy, *GN* Glomerulonephritis, *SBP* systolic blood pressure, *DBP* diastolic blood pressure, *Residual GFR* residual renal glomerular filtration rate, *APD* Automated peritoneal dialysis, * iPTH* intact parathyroid hormone, *LDL* low-density lipoprotein, *HDL* high-density lipoprotein, *ALT* alanine aminotransferase, *AST* aspartate aminotransferase, *hsCRP* high-sensitivity C-reactive protein, *TSAT* transferrin saturation, *SF* serum ferritin

1:1 PSM (based on demographics, hemoglobin, albumin, lipid profiles, and initial urine volume) yielded a balanced cohort of 136 patients (68 per group). All post-matching covariates included in the propensity score model achieved balance, with Standardized Mean Differences (SMDs) < 0.1 (range, 0.011–0.097; Supplementary Table S1), confirming the adequacy of the matching procedure. No patients in either the Roxadustat or rhuEPO group received steroids or other immunosuppressive therapies during the study period.

### Risk of peritoneal dialysis-associated peritonitis

Prior to matching, 296 participants (Roxadustat, *n* = 90; rhuEPO, *n* = 206) contributed 545.99 person years of follow-up. A total of 58 participants developed peritonitis, accounting for 73 episodes overall (IR: 0.134 episodes/person-year, 95% CI: 0.109–0.166). In the unadjusted analysis, the IRs were numerically similar between the groups (Roxadustat: 0.129; rhuEPO: 0.135), and the unadjusted incidence rate ratio (IRR) did not show a significant difference between treatment arms.

Following PSM, the matched cohort of 136 patients contributed 225.85 person-years of follow-up, during which 41 peritonitis episodes occurred in 32 participants (Roxadustat, 12 episodes in 10 patients; rhuEPO, 29 episodes in 22 patients). The crude IR in the matched Roxadustat group was 0.131 episodes per person-year (95%CI, 0.074–0.231), which was substantially lower than the IR in the rhuEPO group (0.212 episodes per person-year; 95%CI, 0.139–0.324). In the propensity-score-matched cohort, univariable Poisson regression analysis demonstrated that compared with the rhuEPO group, the Roxadustat group was associated with a significantly lower incidence of peritonitis, with an IRR of 0.508 (95% CI: 0.249–0.971, *p* = 0.049). No other clinical factors associated with the incidence of peritonitis were identified in the matched cohort by univariable Poisson analysis (Table 2).

**Table 2:** Univariable Poisson regression analysis of peritonitis incident rate ratio in the propensity-score-matched cohort

Variable	β	IRR (95%CI)	p value
Roxadustat vs. rhuEPO	-0.485	0.508(0.249, 0.971)	0.049
Gender, male	-0.233	0.792(0.435, 1.473)	0.451
Age, years	0.013	1.014(0.996, 1.033)	0.147
Diabetes, Yes	0.053	1.055(0.578, 1.929)	0.862
SBP, per 10 mmHg increase	-0.007	0.993(0.916,1.037)	0.807
DBP, per 10 mmHg increase	-0.016	0.984(0.805, 1.195)	0.873
Initial urine volume, per 100mL /24 h change	-0.033	0.967(0.918, 1.061)	0.198
dialysis dose	0.000	1.000(1.000, 1.000)	0.726
Total Kt/V	-0.227	0.797(0.376, 1.615)	0.542
Total Creatinine Clearance	-0.002	0.998(0.987, 1.008)	0.740
Ultrafiltration Volume, ml	0.000	1.000(0.999, 1.000)	0.353
Residual GFR, mL/min	0.015	1.015(0.959, 1.063)	0.569
Residual urine volume, per 100mL /24 h change	0.012	1.012 (0.944–1.085)	0.731
APD vs. Manual PD	-0.530	0.589(0.210, 1.651)	0.314
Serum creatinine, μmol/L	0.001	1.001(1.000, 1.001)	0.188
Serum urea, mmol/L	0.019	1.019(0.993, 1.046)	0.144
Albumin, g/L	-0.048	0.953(0.894, 1.017)	0.139
Hemoglobin, g/L	-0.075	0.928(0.771, 1.097)	0.405
Serum calcium, mmol/L	-0.386	0.679(0.225, 2.091)	0.499
Serum phosphorus, mmol/L	0.056	1.058(0.663, 1.675)	0.813
iPTH, pg/mL	0.016	1.016(0.871, 1.162)	0.829
Total bile acids, umol/L	0.053	1.055(0.968, 1.127)	0.166
Triglycerides, mmol/L	-0.127	0.881(0.587, 1.243)	0.508
Total cholesterol, mmol/L	0.077	1.080(0.806, 1.418)	0.592
LDL, mmol/L	0.013	1.013(0.658, 1.518)	0.950
HDL, mmol/L	0.201	1.233(0.384, 3.632)	0.725
Fasting glucose, mmol/L	-0.066	0.936 (0.804, 1.078)	0.381
ALT, U/L	-0.230	0.795(0.526, 1.137)	0.245
AST, U/L	-0.006	0.994(0.692, 1.353)	0.973
Total Bilirubin, umol/L	-0.124	0.883(0.758, 1.018)	0.100
Direct Bilirubin, umol/L	0.030	1.031(0.706, 1.460)	0.870
Potassium, mmol/L	0.085	1.088(0.707, 1.660)	0.698
Sodium, mmol/L	0.077	1.080(0.990, 1.186)	0.960
Chloride, mmol/L	0.033	1.033(0.977, 1.096)	0.264
hsCRP, mg/L	-0.048	0.953 (0.896, 1.014)	0.131
TSAT, %	0.010	1.010 (0.986, 1.035)	0.430
SF, ug/L	0.000	1.000 (0.999, 1.001)	0.996
Enrollment year	-0.075	0.928 (0.832, 1.035)	0.178
Study period (Phase 2 vs Phase 1)	-0.219	0.803 (0.429, 1.504)	0.493

Initial urine volume and Residual urine volume were scaled per 100 mL/day; therefore, the regression estimate represents the association for each 100 mL/day increase in residual urine volume

*IRR* incidence rate ratio, *CI* confidence interval, *rhuEPO* recombinant Human Erythropoietin, *SBP* systolic blood pressure, *DBP* diastolic blood pressure, *Residual GFR* residual renal glomerular filtration rate, *APD* automated peritoneal dialysis, *iPTH* intact parathyroid hormone, *LDL* low-density lipoprotein, *HDL* high-density lipoprotein, *ALT* alanine aminotransferase, *AST* aspartate aminotransferase, *hsCRP* high-sensitivity C-reactive protein; *TSAT* transferrin saturation, *SF* serum ferritin

**Fig. 2:**
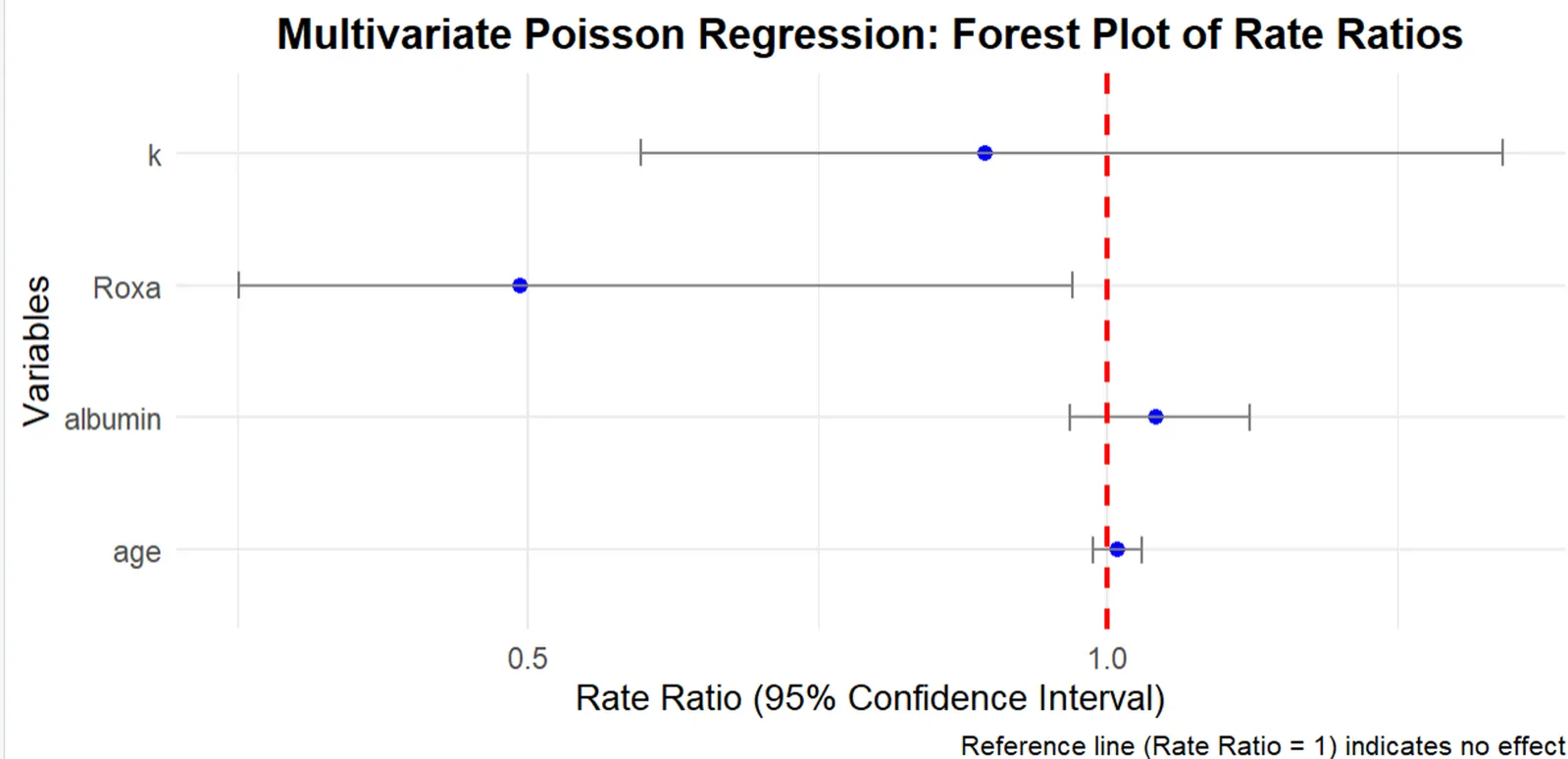
Multivariable Poisson Regression Analysis of Peritonitis Incidence Rate Ratios (IRR) in the Propensity-Score-Matched Cohort of Peritoneal dialysis patients. Peritonitis IRR of Roxadustat treatment as compared to matched rhuEPO treatment control were adjusted for age, serum potassium, and albumin levels. The IRR was calculated as IRR = exp(β), with the 95% confidence interval (CI) derived from the model’s standard errors. *rhuEPO* recombinant Human Erythropoietin, *IRR* incidence rate ratio, *Roxa* Roxadustat treatment, *K* serum potassium

Multivariable Poisson regression demonstrated that Roxadustat treatment was associated with a lower incidence rate of PDAP compared with rhuEPO treatment (IRR, 0.494; 95% CI, 0.252–0.970; *p* = 0.041) after adjusting for age (IRR, 1.008; 95% CI, 0.988–1.029; *p* = 0.430), serum albumin (IRR, 1.042; 95% CI, 0.967–1.122; *p* = 0.278), and serum potassium (IRR, 0.895; 95% CI, 0.597–1.340; *p* = 0.589) (Table 3 and Fig. 2). Further adjusting for PD dose (IRR, 1.000; 95% CI, 1.000–1.000; *p* = 0.783), total creatinine clearance (IRR, 0.999; 95% CI, 0.989–1.010; *p* = 0.923), residual GFR (IRR, 1.003; 95% CI, 0.954–1.055; *p* = 0.910), residual urine volume (IRR1.021,;95% CI,0.952–1.097;*p* = 0.552),diabetes (IRR, 0.819; 95% CI, 0.429–1.564; *p* = 0.545), TSAT(IRR, 1.011; 95% CI, 0.985–1.037; *p* = 0.408) or study phase (phase2 vs. phase1, IRR, 1.384; 95% CI, 0.660–2.904; *p* = 0.390), the association between Roxadustat treatment and lower PDAP incidence remained statistically significant, respectively(all *p* < 0.05) (Table 3). After adjusting for total Kt/V (IRR, 0.838; 95% CI, 0.406–1.731; *p* = 0.633), serum ferritin (IRR, 1.000; 95% CI, 0.998–1.001; *p* = 0.800), or connection system (IRR, 1.548; 95% CI, 0.532–4.504; *p* = 0.422), respectively, statistical significance was attenuated, but the trend of the association between Roxadustat treatment and lower PDAP incidence persisted (Table 3).

**Table 3:** Multivariable Poisson regression analyses of the association between Roxadustat use and peritoneal dialysis-associated peritonitis incidence in the propensity-score-matched cohort

Model	Additional adjustment	IRR for roxadustat	95% CI	p value
Model 1	None	0.494	0.252–0.970	0.041
Model 2	PD dose	0.494	0.252–0.969	0.040
Model 3	Total Kt/V	0.511	0.256–1.019	0.057
Model 4	Total Ccr	0.497	0.250–0.987	0.046
Model 5	Residual GFR	0.496	0.251–0.984	0.045
Model 6	Residual urine volume	0.483	0.245-0.952	0.036
Model 7	Diabetes	0.495	0.252–0.970	0.041
Model 8	TSAT	0.486	0.244–0.967	0.040
Model 9	Serum ferritin	0.509	0.255–1.015	0.055
Model 10	Connection system	0.523	0.264–1.036	0.063
Model 11	Study phase	0.406	0.184–0.899	0.026

Incidence rate ratios (IRRs) were estimated using multivariable Poisson regression. The IRRs, 95% CIs, and p values for Roxadustat are presented separately

Model 1 included treatment group (with rhuEPO as the reference group), age, serum albumin, and serum potassium. Models 2–11 were constructed by adding the listed covariate to Model 1, respectively

For connection system, automated cycler system was used as the reference category. For study phase, Phase 1 was used as the reference category

Residual urine volume was scaled per 100 mL/day

*rhuEPO* recombinant human erythropoietin, *IRR* incidence rate ratio, *CI* confidence interval, *PD* peritoneal dialysis, *PDAP* peritoneal dialysis-associated peritonitis, *Kt/V* urea clearance index, *Ccr* creatinine clearance, *GFR* glomerular filtration rate, *TSAT* transferrin saturation

In a subgroup analysis restricted to patients enrolled during the phase 2 within the matched cohort (rhuEPO, *n* = 21; Roxadustat, *n* = 68), the lower peritonitis incidence rate associated with Roxadustat persisted (crude IRR 0.44, 95% CI 0.20–0.96, *p* = 0.038); additional adjustment for baseline hemoglobin yielded essentially the same estimate (adjusted IRR 0.43, 95% CI 0.19–0.95, *p* = 0.036; Supplementary Tables S2 and S3, Figure S1).

## Discussion

In this propensity-matched cohort, Roxadustat-treated PD patients exhibited a significantly lower peritonitis incidence compared to those receiving rhuEPO.

Findings from prior clinical studies help contextualize our results. Although a recent systematic review in chronic kidney disease (CKD) populations noted higher rates of systemic infections such as sepsis and cellulitis with Roxadustat, the risk of peritonitis was unchanged [19]. Large Phase 3 trials likewise showed similar overall infection rates between Roxadustat and rhuEPO [20]. In early trials specifically involving PD patients, peritonitis emerged as a frequent serious adverse event, yet investigators consistently judged these episodes to be unrelated to Roxadustat and none required treatment discontinuation [21, 22]. These observations suggest that Roxadustat is at least as safe as rhuEPO with respect to peritonitis, supporting the relevance of our findings in a real-world PD setting.

Consistent with multicenter findings by Zhang et al. [6], our single-center study confirmed the association between Roxadustat and reduced peritonitis risk, while offering several methodological refinements. First, unlike Zhang et al.‘s Cox regression—which modeled time to first peritonitis event and censored patients thereafter—we applied multivariable Poisson regression to capture recurrent episodes and total person-years of exposure, yielding a more comprehensive Incidence Rate Ratio (IRR). Second, whereas Zhang et al. permitted prior ESA exposure with only a one-week washout, our strict “first medication” principle enrolled exclusively treatment-naïve patients on monotherapy, minimizing carry-over effects and eliminating biases from prior ESA exposure or crossover. Finally, our single-center setting ensured uniform PD training and diagnostic criteria, with follow-up extending through 2024 providing updated real-world evidence. Together, these refinements enabled a more comprehensive assessment and showed an association between Roxadustat use and a lower peritonitis incidence rate in PD patients, although this finding should be interpreted cautiously given the observational design and limited number of events.

We speculate that the observed lower incidence of PDAP in the Roxadustat group compared to rhuEPO may be attributed to Roxadustat’s effects mediated by HIF stabilization, which differ fundamentally from rhuEPO’s erythropoietin receptor activation. Unlike rhuEPO therapy that often necessitates high intravenous iron doses [23, 24], Roxadustat enhances iron metabolism by increasing transferrin and total iron-binding capacity while suppressing hepcidin [25]. This mechanism may reduce inflammation-related functional iron deficiency common in PD patients, thereby supporting immune cell function. Given that SF is a potential indicator of iron status and inflammation, it warrants consideration as a possible confounder. Notably, although baseline SF levels were lower in the Roxadustat group after matching (median: 129.00 vs. 185.80 µg/L, *p* = 0.021), both values fall within the target clinical range, and univariate analysis showed no significant association between SF and peritonitis risk, suggesting this difference is unlikely to affect our primary outcome. Chronic inflammation and oxidative stress contribute to peritoneal membrane injury in CKD and PD [26–28]. Roxadustat’s correction of anemia despite elevated inflammatory markers suggests that HIF activation confers anti-inflammatory and antioxidant effects, potentially protecting peritoneal membrane integrity [29, 30]. Finally, we speculate that HIF stabilization might support epithelial and endothelial barrier integrity [31], potentially strengthening the peritoneal mesothelial barrier against microbial translocation.

Key strengths of this study include the use of PSM to minimize baseline confounding and a strict monotherapy inclusion criterion to ensure medication-specific observations. However, the single-center retrospective design may limit generalizability and introduce potential unmeasured confounding. Key variables—such as hygiene practices, invasive procedure frequency, and iron supplementation dosage—were not systematically recorded at the individual level and therefore could not be fully accounted for in the analysis. We must also acknowledge that the limited sample size and small number of peritonitis episodes restricted our multivariate analysis of multiple potential risk factors for peritonitis. Although additional sensitivity analyses incorporating dialysis adequacy indices and other clinically relevant variables showed directionally consistent, statistical significance was attenuated in some models. Therefore,the observed association should be interpreted cautiously and cannot establish a causal effect. Additionally, although our trend analysis showed no significant association between enrollment period and peritonitis risk, temporal bias and a “period effect”—stemming from the sequential introduction of rhuEPO and Roxadustat (pre- and post-2019)—cannot be entirely excluded, as decade-long advancements in PD techniques and infection control may influence peritonitis incidence [32, 33]. Future prospective multicenter studies are needed to validate these findings.

## Conclusion

Roxadustat treatment was observed to be associated with a reduced incidence of PDAP as compared to their matched control treated by rhuEPO. This finding suggests that the pleiotropic effects of Roxadustat, may be mediated by HIF stabilization. However, the validity of this effect and underlying mechanism requires confirmation through larger-scale prospective studies.

## Supplementary Information

Below is the link to the electronic supplementary material.

Supplementary Material 1

## Data Availability

The data supporting the findings of this study are available from the corresponding author, Wen Tang, upon reasonable request.
