# Supplementary material for: Roxadustat and incidence of peritoneal dialysis-associated peritonitis: a propensity score–matched cohort study
Source: Clin Exp Nephrol. 2026 Aug 10;30(11):1619–27. doi: 10.1007/s10157-026-02926-6 (PMC13668977; doi:10.1007/s10157-026-02926-6)
Supplement: Supplementary file 1 [file 10157_2026_2926_MOESM1_ESM.doc]

Supplementary Table S1. Covariate balance in the main propensity score matching model.

| **Variable** | **Before matching** | | | **After matching** | | | **Balance after matching** |
| --- | --- | --- | --- | --- | --- | --- | --- |
|  | **Roxadustat** | **rhuEPO** | **SMD** | **Roxadustat** | **rhuEPO** | **SMD** |  |
| Age | 60.23 | 57.43 | +0.168 | 59.03 | 59.55 | −0.032 | Balanced |
| Male sex | 0.733 | 0.621 | +0.241 | 0.721 | 0.691 | +0.065 | Balanced |
| Baseline albumin | 34.60 | 37.55 | −0.591 | 35.47 | 35.33 | +0.031 | Balanced |
| Baseline hemoglobin | 92.91 | 87.19 | +0.332 | 91.24 | 91.06 | +0.011 | Balanced |
| Initial urine volume, mL/24 h | 1122.89 | 1281.07 | −0.242 | 1134.41 | 1182.79 | −0.072 | Balanced |
| Triglycerides | 1.43 | 1.61 | −0.228 | 1.50 | 1.47 | +0.031 | Balanced |
| Total cholesterol | 3.53 | 4.32 | −0.707 | 3.67 | 3.75 | −0.082 | Balanced |
| LDL cholesterol | 1.96 | 2.58 | −0.721 | 2.04 | 2.11 | −0.097 | Balanced |
| HDL cholesterol | 0.85 | 1.03 | −0.604 | 0.89 | 0.89 | −0.020 | Balanced |

Note: Balance was assessed using standardized mean differences (SMDs). Signed SMDs are reported, calculated as the mean (or proportion) in the Roxadustat group minus that in the rhuEPO group and divided by the pooled standard deviation; positive values therefore indicate higher values or proportions in the Roxadustat group, whereas negative values indicate lower values or proportions in the Roxadustat group. An absolute SMD < 0.10 was considered indicative of acceptable balance. The main propensity score matching model incorporated age, sex, baseline albumin, baseline hemoglobin, residual urine volume, triglycerides, total cholesterol, LDL cholesterol, and HDL cholesterol. This model generated 68 matched pairs. After matching, all covariates included in the propensity score model achieved acceptable balance (range of absolute SMDs, 0.011–0.097).

*Abbreviations:* SMD, standardized mean difference; rhuEPO, recombinant human erythropoietin; LDL, low-density lipoprotein; HDL, high-density lipoprotein.

Supplementary Table S2. Covariate balance within the post-marketing subgroup (Sep 2019 - Sep 2024)

| Variable | rhuEPO (n=21) | Roxadustat (n=68) | Absolute SMD |
| --- | --- | --- | --- |
| n | 21 | 68 |  |
| Age, years | 58.61 (17.81) | 59.03 (16.20) | 0.025 |
| Female sex, n (%) | 7 (33.3) | 19 (27.9) | 0.117 |
| Serum albumin, g/L | 35.21 (3.78) | 35.47 (4.58) | 0.062 |
| Hemoglobin, g/L | 94.86 (15.43) | 91.24 (16.16) | 0.229 |
| Initial urine volume, mL/24 h | 1073.81 (582.66) | 1134.41 (679.72) | 0.096 |
| Triglycerides, mmol/L | 1.46 (0.73) | 1.50 (0.75) | 0.044 |
| Total cholesterol, mmol/L | 3.63 (0.95) | 3.67 (1.00) | 0.042 |
| LDL-C, mmol/L | 1.93 (0.65) | 2.04 (0.79) | 0.151 |
| HDL-C, mmol/L | 0.87 (0.28) | 0.89 (0.26) | 0.087 |

Note: Covariate balance between treatment groups within the post-marketing subgroup (September 2019 – September 2024; n = 89). Restricting the matched cohort to the post-marketing period disrupted the original 1:1 matching, yielding an unmatched 21 vs 68 subset; covariate balance was therefore re-assessed in this subgroup. Continuous variables are presented as mean (SD) and categorical variables as n (%). Standardized mean differences (SMDs) are reported for each covariate, with an SMD < 0.2 considered acceptable. Most covariates remained within this range; hemoglobin showed the largest between-group difference (SMD = 0.23). Abbreviations: rhuEPO, recombinant human erythropoietin; SD, standard deviation; SMD, standardized mean difference; LDL-C, low-density lipoprotein cholesterol; HDL-C, high-density lipoprotein cholesterol.

Supplementary Table S3. Peritonitis incidence rate and rate ratios in the post-marketing subgroup

| Group | N | Events | Person-years | Rate (per p-y) | Crude IRR (95% CI) | Crude P | Hgb-adjusted IRR (95% CI) | Adjusted P |
| --- | --- | --- | --- | --- | --- | --- | --- | --- |
| rhuEPO (reference) | 21 | 13 | 47.8 | 0.272 | Reference |  | Reference |  |
| Roxadustat | 68 | 12 | 101.4 | 0.118 | 0.44 (0.20-0.96) | 0.038 | 0.43 (0.19-0.95) | 0.036 |

Note: Peritonitis incidence rate and incidence rate ratios (IRR) in the post-marketing subgroup (September 2019 – September 2024). The crude IRR was estimated by Poisson regression with the logarithm of person-years as an offset, comparing Roxadustat with rhuEPO (reference). The hemoglobin-adjusted IRR additionally adjusted for baseline hemoglobin, the only covariate exceeding the prespecified SMD threshold of 0.20 in Supplementary Table S2. IRR = exp(β); 95% confidence intervals were derived from model standard errors. Abbreviations: IRR, incidence rate ratio; CI, confidence interval; rhuEPO, recombinant human erythropoietin; p-y, person-years.

**Figure S1.** Forest plot of peritonitis incidence rate ratios (Roxadustat vs rhuEPO) in the post-marketing subgroup (September 2019 – September 2024; n = 89), visualizing the estimates reported in Table S3. Results are shown for the crude Poisson model and the hemoglobin-adjusted sensitivity analysis. Points denote the IRR and horizontal bars the 95% confidence interval; the red dashed line at IRR = 1 indicates no effect, and the x-axis is on a logarithmic scale. Both models yielded consistent estimates (crude IRR 0.44, 95% CI 0.20–0.96, P = 0.038; hemoglobin-adjusted IRR 0.43, 95% CI 0.19–0.95, P = 0.036).
